# Hydrodissection of Wiltse’s Plane to Facilitate Exposure During Minimally Invasive Transforaminal Lumbar Interbody Fusion

**DOI:** 10.7759/cureus.1872

**Published:** 2017-11-23

**Authors:** Zachary Tataryn, Kenan Alkhalili, James T Kryzanski

**Affiliations:** 1 Neurosurgery, The Ottawa Hospital; 2 Neurosurgery, Cairo University; 3 Department of Neurosurgery, Tufts Medical Center

**Keywords:** surgical exposure, spondylolisthesis, tlif, ultrasound

## Abstract

Traditional posterior lumbar approaches in a transforaminal lumbar interbody fusion (TLIF) require subperiosteal dissection of bilateral paraspinal muscles to provide adequate exposure. This may traumatize the multifidus muscle and its afferent innervations leading to postoperative paraspinal muscle atrophy. Minimizing such intraoperative trauma has been identified as an important factor in the reduction of postoperative lumbar pain. An approach via a blunt dissection through Wiltse’s plane, which lies between the longissimus and multifidus muscles, may minimize postoperative pain. Definition of this plane may be facilitated by local injection of 1% lidocaine within the plane itself, as well as in the musculature defining its borders.

In this paper, we demonstrate this technique with a 55-year-old female patient who presented with left-sided radicular leg pain in an L5 distribution. Wiltse plane hydrodissection was utilized in performing an L4-5 TLIF. Ultrasound images of the patient’s sub-fascial musculature were obtained pre- and posthydrodissection to assess the elucidation of this plane through this technique. Intraoperative images were obtained following dissection of Wiltse’s plane to further illustrate the facilitation of exposure of Wiltse’s plane through hydrodissection. Postoperatively the patient did well citing a complete resolution of her radicular pain. She did not require intravenous (IV) pain medication, as her postoperative pain was well controlled with oral pain medication. She was mobilized on post-op day one, and discharged home on post-op day two with minimal back pain.

Our initial experience supports the feasibility, safety, and effectiveness of hydrodissection of Wiltse’s plane to facilitate exposure during a minimally invasive TLIF and thereby reducing postoperative pain.

## Introduction

Traditional posterior lumbar approaches in a transforaminal lumbar interbody fusion (TLIF) require subperiosteal dissection of bilateral paraspinal muscles to provide adequate exposure. This may traumatize the multifidus muscle and its afferent innervations leading to postoperative paraspinal muscle atrophy, which has been identified as an important factor in postoperative lumbar pain [1,2].

An approach via a blunt dissection through Wiltse’s plane, which lies between the longissimus and multifidus muscles, avoids a traditional subperiosteal dissection, and has been termed as the mini-open approach in performing a TLIF [3]. Treatment via both traditional and mini-open approaches appears to have similar clinical efficacies, however, the mini-open approach has been shown to reduce the postoperative incidence of chronic low back pain [4]. The Wiltse approach is also widely utilized in the treatment of thoracic/lumbar vertebral body fractures, lateral/far lateral lumbar disc herniation, lumbar lateral recess stenosis, as well as other spinal pathologies [5-8].

**Figure 1:**
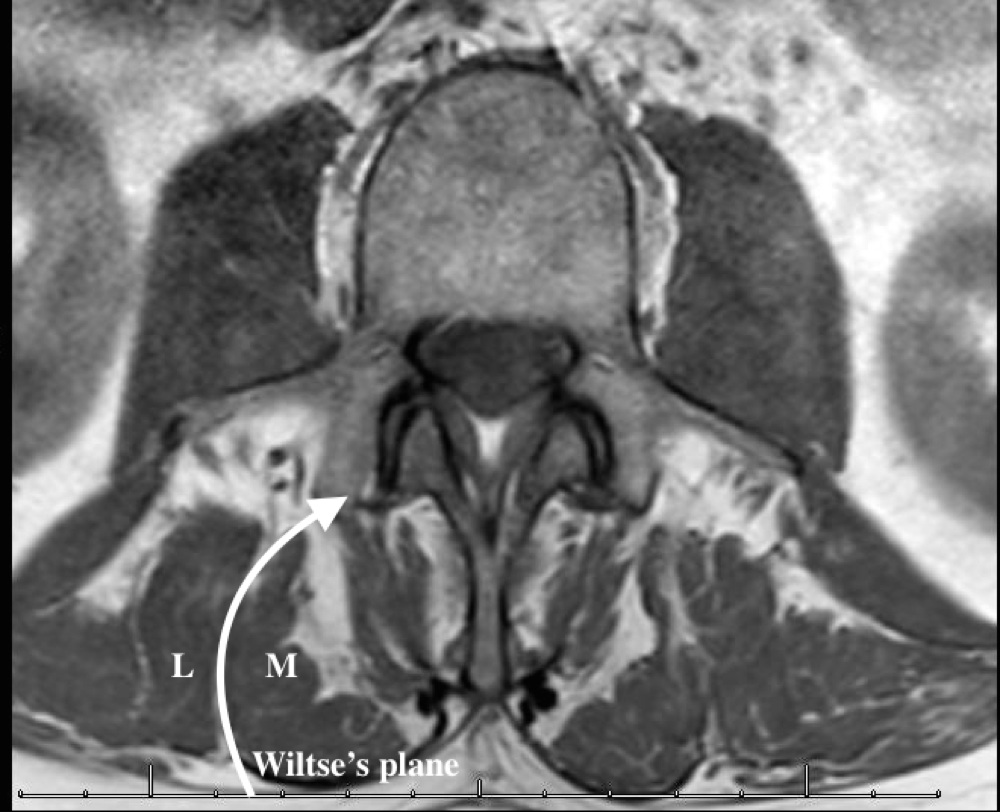
Axial magnetic resonance imaging (MRI) slice illustrating Wiltse's plane. Axial MRI revealing Wiltse’s plane separating the multifidus muscle (M) from the longissimus muscle (L).

Posterior spinal erector musculature is primarily composed of the longissimus dorsi and multifidus muscles. In the lumbar region, the longissimus dorsi originates from the dorsal surface of all transverse processes and the anterior layer of the lumbodorsal fascia, and inserts at the tips of the spinous processes of the thoracic vertebrae and lower nine or ten ribs (between the tubercle and the angle). The longissimus functions to both extent and laterally flexes the lumbar spine. Lumbar multifidus muscles originate from vertebral mamillary processes as a thin fasciculus, then extend rostrally and medially to insert at the spinous processes of rostral vertebrae (up to three above). The multifidus plays a key role in stabilizing the joints between adjacent vertebral bodies. Wiltse’s plane is clearly visualized on magnetic resonance imaging (MRI) (Figure 1), however, it can prove challenging to locate intraoperatively.

Due to varying patient anatomy, identification of Wiltse’s plane can at times prove challenging. In this paper, we describe a novel and simple technique of utilizing local injection of 1% lidocaine into both longissimus and multifidus muscles, thereby facilitating the identification and dissection of Wiltse’s plane via hydro-dissection.

## Technical report

The patient utilized to demonstrate this technique is a 55-year-old woman who presented in our clinic with a six-month history of severe left leg pain in an L5 distribution that was refractory to medical management and physical therapy. Her lumbar imaging studies revealed severe lumbar spinal stenosis from L3 through L5, as well as an unstable L4-5 spondylolisthesis with impingement of her left L5 nerve root. The patient consented to decompressive laminectomies from L3 to L5 (bilateral decompression through a minimally invasive unilateral approach on the left), a left L4-5 TLIF, and bilateral pedicle screw fixation at L4-5. In addition, she consented to our filming the procedure, and the use of ultrasonography to illustrate the efficacy of the aforementioned hydro-dissection of Wiltse’s plane.

General endotracheal anesthesia was induced in the usual fashion, and the patient was placed prone onto gel rolls. We used lateral fluoroscopy to localize an incision centered over the L3-L5 area on the left and the L4-L5 area on the right. We infiltrated the incisional areas with lidocaine and epinephrine. Each incision was approximately 35 mm lateral to midline. We first used a #10 blade to make the left-sided incision to the subcutaneous tissue. We used monopolar electrocautery to dissect the lumbodorsal fascia and open it.

At this point, 10cc of 1% lidocaine with 1:200,000 units of epinephrine was injected via numerous injections along the entirety of the medial edge of the muscle thought to be the multifidus (along the medial edge of Wiltse’s plane). These injections were performed during the withdrawal of the syringe, in attempt to deposit the lidocaine deep into the desired plane. 10cc was then injected into the presumed longissimus muscle in a similar fashion.

**Figure 2:**
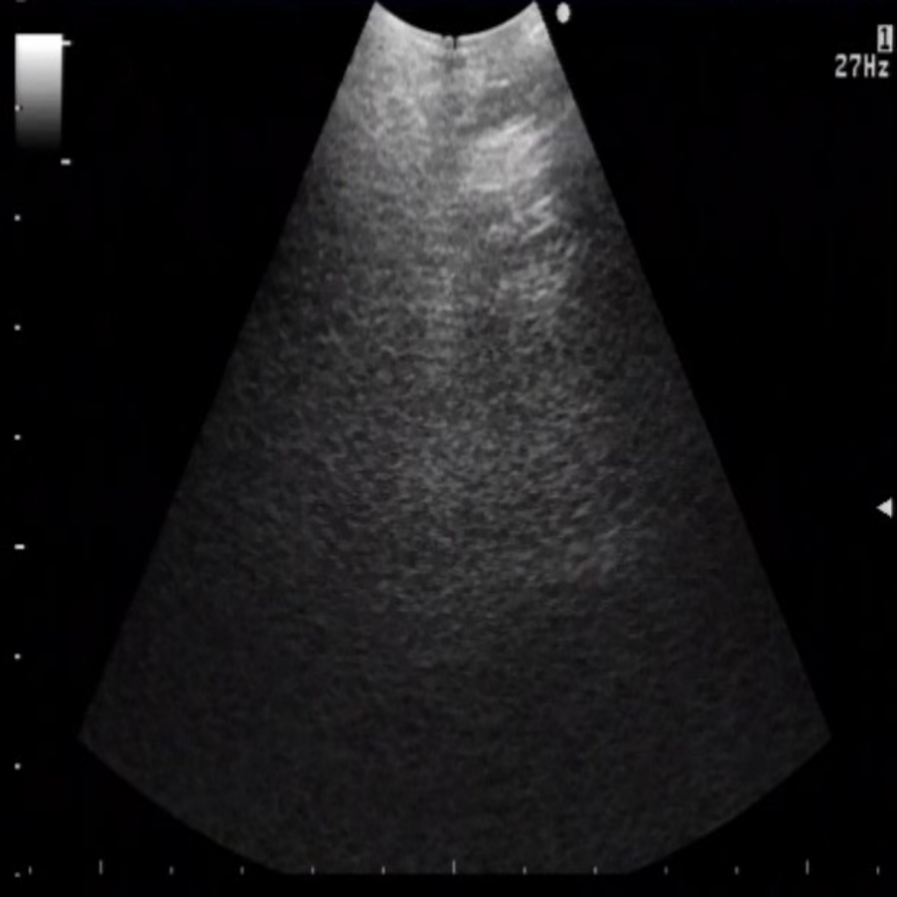
Axial ultrasound imaging: pre-hydrodissection.

**Figure 3:**
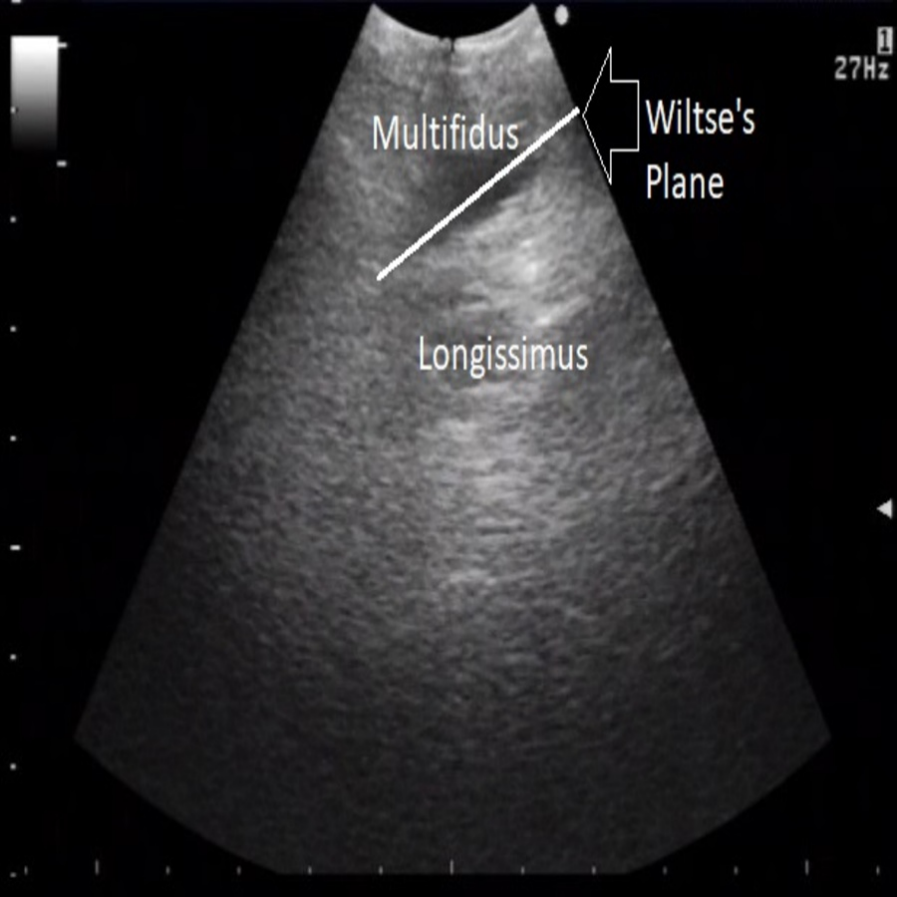
Axial ultrasound imaging: post-hydrodissection. The white line reveals Wiltse’s plane, and separates the multifidus muscle medially from the longissimus muscle laterally.

**Figure 4:**
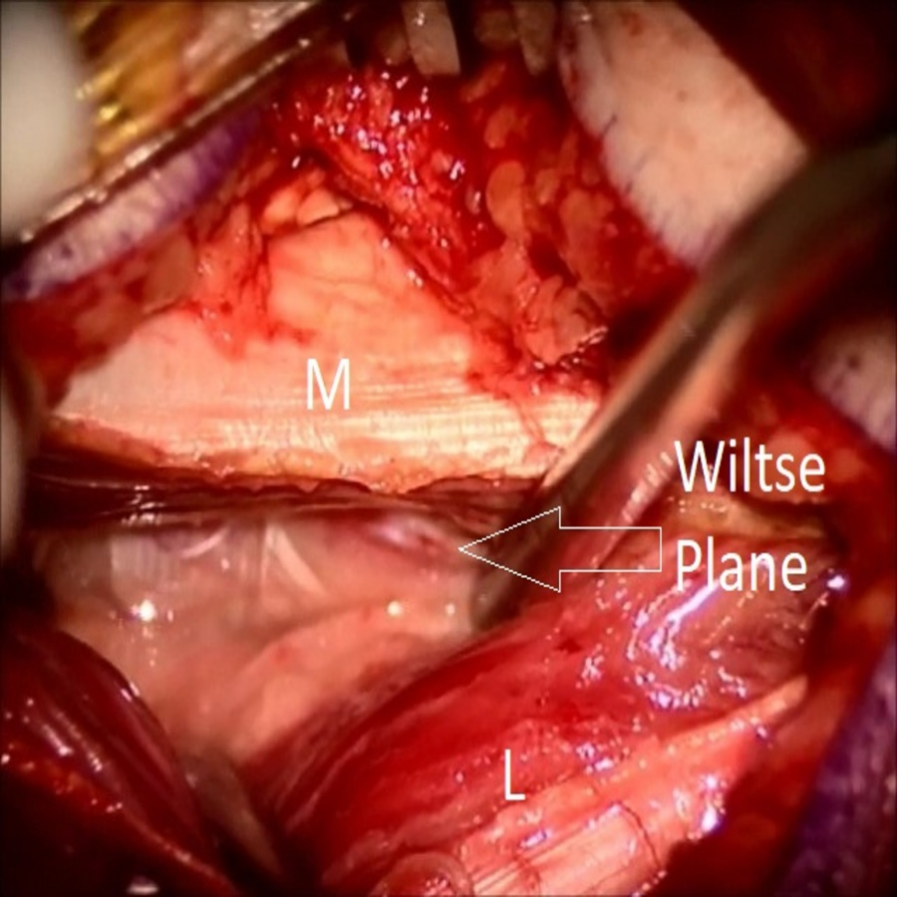
View from operative microscope of hydrodissection plane. View from operative microscope of hydrodissection plane. The multifidus (M) is cleanly separated from the longissimus dorsi (L).

Ultrasound images of Wiltse’s plane, pre and postinjection, were obtained (Figures 2, 3), demonstrating an effective hydro-dissection of the planned trajectory. Wiltse’s plane, now clearly exposed, was separated with ease utilizing a Penfield 4 (Figure 4). The remainder of the procedure went as planned and without complication. Postoperatively the patient did well citing a complete resolution of her radicular pain. She did not require intravenous (IV) pain medication, as her postoperative pain was well controlled with oral pain medication. She was mobilized on post-op day one, and discharged home on post-op day two citing minimal back pain.

## Discussion

The Wiltse approach to the lumbar spine was first described in 1968, and utilizes an incision approximately 4.5 cm lateral to the midline to gain access to the lower lumbar and sacral vertebrae through a natural cleavage plane between the multifidus and longissimus dorsi muscles [9]. Following Wiltse’s plane provides direct access to the transverse process, pars, and facet joint with minimal soft tissue retraction [10]. It has been postulated that this approach reduces postoperative pain due to both minimized soft tissue distraction/dissection, as well as the preservation of the supraspinous and interspinous ligaments [9]. In addition, an approach via Wiltse’s plane is less vascular than a mid-line approach, which results in less intraoperative bleeding [11].

Intraoperative localization of Wiltse’s plane can prove difficult at times due to varying patient anatomy. Wiltse’s plane has been noted to have a variation of 2.4-7 cm from the midline in cadaveric studies [12, 13]. It has been suggested that preoperative MRI imaging may facilitate the planning of skin incision to more precisely localize Wiltse’s plane given this varying patient anatomy [10].

We report a novel and simple method of exposure/dissection of Wiltse’s plane in transverse approaches to the lumbar spine. This technique involves the injection of local anesthetic agents both in between and directly into the muscles bordering Wiltse’s plane: the longissimus (lateral border) and multifidus (medial border). This technique allows for easy visualization of Wiltse’s plane by dilating the bodies of the injected muscles, making the plane concealed at their junction more pronounced. In addition, injections into the plane itself effectively hydrodissect the muscles apart from each other. This minimizes damage to intrinsic spine musculature caused by aggressive dissection, thereby reducing postoperative pain [2, 9].

## Conclusions

Traditional posterior lumbar approaches in a TLIF require subperiosteal dissection of bilateral paraspinal muscles to provide adequate exposure. This may traumatize the multifidus and longissimus muscles and their afferent innervations leading to postoperative paraspinal muscle atrophy. An approach via a blunt dissection through Wiltse’s plane, which lies between the longissimus and multifidus muscles, may minimize postoperative pain. Definition of this plane may be facilitated by local injection of 1% lidocaine within the plane itself, as well as in the musculature defining its borders. Our initial experience supports the feasibility, safety, and effectiveness of hydrodissection of Wiltse’s plane to facilitate exposure during a minimally invasive TLIF and thereby reducing postoperative pain.
